# State of the Science of Dementia Care for Asian Americans: Integrative Review

**DOI:** 10.1093/geroni/igaf122.2981

**Published:** 2025-12-31

**Authors:** Jiyoon Jang, Kalisha Bonds Johnson, Glenna Brewster, Mi-Kyung Song

**Affiliations:** Emory University, Atlanta, Georgia, United States; Emory University, Atlanta, Georgia, United States; Emory University, Atlanta, Georgia, United States; Emory University, Atlanta, Georgia, United States

## Abstract

Asian Americans account for >8% of persons living with Alzheimer’s disease and related dementia (ADRD, PLwD) despite being < 6% of the nation’s population. Many are cared for at home by family members or relatives, but the literature is sparse about their specific caregiving experiences. We conducted an integrative review of the existing literature to examine the extent of dementia caregiving research in this population, including study characteristics and empirical evidence of caregiving experiences. We searched PubMed, CINAHL, PsycINFO, Embase, and Web of Science for reports of studies of Asian American caregivers of PLwD conducted in the U.S. published in English between 1999 and 2024. The final sample included 35 articles: of which, 51.4% (n = 18) were conducted in California, and 51.4% (n = 18) were qualitative studies using semi-structured interviews and focus groups. Chinese and Korean caregivers were most frequently studied (14 and 12 articles, respectively) followed by Vietnamese caregivers. Seventeen (48.6%) articles reported a negative impact of caregiving on mental health, e.g., depression, burden, and anxiety. Caregiving challenges included limited access to culturally competent health and community services, distance from home, costs, and stigma. Caregiver intervention studies, (n = 8, 22.9%) including cognitive strategies, self-care, and religious practices demonstrated mixed outcomes in improving caregiver outcomes. Studies predominantly focused on negative outcomes of caregiving. Current evidence suggests that caregiving has a negative impact on the psychological health of Asian American caregivers. However, additional research is needed to examine positive effects of caregiving or more balanced perspectives and experiences of caregiving in this population.

